# Real and hypothetical monetary rewards modulate risk taking in the brain

**DOI:** 10.1038/srep29520

**Published:** 2016-07-07

**Authors:** Sihua Xu, Yu Pan, You Wang, Andrea M. Spaeth, Zhe Qu, Hengyi Rao

**Affiliations:** 1 Laboratory of Applied Brain and Cognitive Sciences, School of Business and Management, Shanghai International Studies University, Shanghai, China; 2 Department of Psychology, Sun Yat-sen University, Guangzhou, China; 3 Department of Applied Psychology, Guangdong University of Finance and Economics, Guangzhou, China; 4 Center for Functional Neuroimaging, Department of Neurology, Perelman School of Medicine, University of Pennsylvania, Philadelphia, PA, USA; 5 School of Public Health and Tropical Medicine, Department of Psychology, Southern Medical University, Guangzhou, China

## Abstract

Both real and hypothetical monetary rewards are widely used as reinforcers in risk taking and decision making studies. However, whether real and hypothetical monetary rewards modulate risk taking and decision making in the same manner remains controversial. In this study, we used event-related potentials (ERP) with a balloon analogue risk task (BART) paradigm to examine the effects of real and hypothetical monetary rewards on risk taking in the brain. Behavioral data showed reduced risk taking after negative feedback (money loss) during the BART with real rewards compared to those with hypothetical rewards, suggesting increased loss aversion with real monetary rewards. The ERP data demonstrated a larger feedback-related negativity (FRN) in response to money loss during risk taking with real rewards compared to those with hypothetical rewards, which may reflect greater prediction error or regret emotion after real monetary losses. These findings demonstrate differential effects of real versus hypothetical monetary rewards on risk taking behavior and brain activity, suggesting a caution when drawing conclusions about real choices from hypothetical studies of intended behavior, especially when large rewards are used. The results have implications for future utility of real and hypothetical monetary rewards in studies of risk taking and decision making.

Understanding how people take risks and make decisions is a challenge for many disciplines such as economics, psychology, and neuroscience. Individual decisions are often motivated by the simulation of rewards. As a secondary but strong incentive, monetary reward is widely used in the real-world situations as well as in laboratory studies. In the real-world, companies typically use real money to retain and motivate their workers^1^. However, in laboratory studies, monetary rewards are often hypothetical due to the impractical expensive cost and/or unethical issues associated with real money incentives. In order to draw conclusions about real choices from hypothetical reports of intended behavior, it is necessary to validate that real and hypothetical rewards have a similar effect for guiding people’s action choice.

Various cognitive and economical paradigms have been used to compare the effects of real versus hypothetical monetary rewards on risk taking and decision making behavior, including the discounting task (DDT)^2^,^3^,^4^,^5^,^6^, probability discounting task (PDT)^7^,^8^,^9^, social discounting task^10^, Iowa Gambling Task (IGT)^11^, and economical games like the sharing game, the ultimatum game, and the dictator game^12^. However, the findings from these studies are inconsistent. While some studies showed similar effects^2^,^3^,^4^,^5^,^7^,^10^,^11^, other studies reported significant differential effects of real versus hypothetical rewards on decision behavior^6^,^8^,^9^,^12^. Moreover, people may overstate hypothetical valuations and plans compared to real choices, suggesting a “hypothetical bias” during decision making^13^,^14^,^15^,^16^,^17^. Using function magnetic resonance imaging (fMRI), neuroimaging studies have also reported inconsistent findings regarding the effects of real versus hypothetical monetary rewards on brain activation patterns during decision making. No differences in brain activation were observed between real versus hypothetical monetary rewards during discounting tasks in one study^18^; however, another study found stronger activity in brain regions in responses to the value signals during a real choice condition compared to hypothetical choice condition^19^.

The balloon analogue risk task (BART) has also been used to examine the effects of real and hypothetical monetary rewards on risk taking behavior^20^. The BART is laboratory-based cognitive task originally developed by Lejuez and colleagues^21^ to provide an ecologically valid model for the assessment of risk taking propensity and behavior. During this task, participants are repeatedly given the option to continue or discontinue inflating a virtual balloon that could growing larger or explode (see Fig. 1). A larger balloon is associated with an increased probability of explosion but also with the potential for greater reward. Unlike other paradigms such as the IGT in which risk is defined and manipulated by arbitrary connections between stimuli and outcomes, risk in the BART is more directly and ecologically defined as the probability of explosion for each balloon. The average number of inflation pumps participants made for the balloons provides an objective assessment of risk taking propensity^20^,^21^. When comparing real versus hypothetical monetary rewards using the BART, previous studies have shown that subjects stop inflating balloons earlier and are more risk averse for a balloon if they received a negative outcome (i.e., money loss) during the previous balloon trial in the real reward monetary condition^20^. However, the neural mechanisms underlying the differential effects of real versus hypothetical monetary rewards on risk taking and decision making behavior remain unknown.

In the present study, we used electroencephalography (EEG) to measure time-locked event-related potential (ERP) responses to risky decision making during the BART, in order to compare the effects of real and hypothetical rewards on risk taking behavior and brain activity. ERP is a non-invasive electrophysiological measure of brain response consisting of a series of positive and negative voltage deflections that is the direct result of a specific sensory, motor, or cognitive process^22^. Previous decision making studies have used EEG and identified a specific ERP component, the feedback-related negativity (FRN), which is sensitive to feedback valence and typically more pronounced for negative outcomes during decision making^23^,^24^,^25^. We hypothesized that BART risk taking would show larger FRN in response to negative feedback (i.e., money loss due to balloon explosion) during real monetary reward condition as compared to hypothetical reward condition.

## Results

### Behavior Results

When averaging across all balloon trials, subjects tended to pump the balloon more times in the hypothetical reward condition than in the real reward condition (mean ± SD, 7.70 ± 1.40 vs. 7.18 ± 1.13, *t*(18) = 1.89, *p* = 0.07, *Cohen’s d* = 0.41; Fig. 2a). Further analysis showed that, during the trials immediately following a negative feedback, subjects pumped the balloon significantly more times in the hypothetical reward condition than in the real reward condition (7.72 ± 1.52 vs. 7.08 ± 1.35, *t*(18) = 2.65, *p* = 0.016, *Cohen’s d* = 0.45; Fig. 2b left). However, during the trials immediately following a positive feedback, the number of balloon pumps did not differ between the hypothetical and real reward conditions (7.74 ± 1.46 vs. 7.35 ± 1.20, *t*(18) = 0.99, *p* > *0.3*, Fig. 2b right).

### ERP Results

In line with previous ERP studies of the BART^26^,^27^,^28^, an obvious FRN component was observed at the Cz electrode site (Fig. 3a,c). Therefore, we analyzed the FRN using the mean amplitudes of ERP responses to different trials in the 180–240 ms time window post-onset of feedback from the Cz location. There were significantly more negative ERP responses to balloon loss (negative feedback) during the real reward condition compared to the hypothetical reward condition (5.12 ± 5.03 μV vs. 8.44 ± 6.88 μV, *t*(18) = −3.58, *p* = 0.002, *Cohen’s d* = 0.56; Fig. 3b). However, no differences were found between FRN amplitudes in response to balloon win (positive feedback) in the real reward condition compared to those in the hypothetical reward condition (5.99 ± 3.47 μV vs. 6.61 ± 3.13 μV, *t*(18) = −0.85, *p* > 0.4, Fig. 3b). A 2 (valence: positive/negative) × 2 (reward type: real/hypothetical) repeated measures ANOVA analysis on the FRN amplitudes (Fig. 2b) revealed a significant interaction between reward type and feedback valence (F(1, 18) = 5.41, *p* = 0.032, *η*^2^ = 0.23), a significant main effect of the reward type (F(1, 18) = 10.86, *p* = 0.004, *η*^2^ = 0.38), and no main effect of feedback valence (F(1, 18) = 0.26, *p* > 0.60, *η*^2^ = 0.01). The different waveforms between the negative feedback and positive feedback are displayed in Fig. 3c, which show a greater FRN component for the real reward condition than the hypothetical reward condition. However, no correlations were observed between the FRN amplitudes and risk taking behavior during the BART for both real and hypothetical reward conditions (all *p* > 0.05).

## Discussion

In order to determine whether real and hypothetical monetary rewards modulate risk taking behavior and brain function in a similar manner, the present study used ERP and examined both behavioral and brain responses to positive and negative outcomes from increased risk taking in a BART paradigm. Behavioral data showed that subjects tended to inflate the balloon fewer times in the real monetary reward condition compared to the hypothetical monetary reward condition, especially after negative feedback of balloon burst (money loss). Furthermore, ERP data demonstrated a larger FRN response after a loss trial (balloon explosion and money loss) in the real reward condition compared to the hypothetical reward condition. These findings suggest that real and hypothetical monetary rewards have differential effects on risk taking behavior and brain activity.

The behavioral data in this study are consistent with findings from our previous study showing greater risk aversion during balloon inflation with real monetary rewards^20^. The results are also in line with previous studies reporting less impulsive choices associated with real monetary rewards^8^,^9^. However, these findings are inconsistent with those studies that failed to detect differences in choices behavior using discounting and gambling paradigms^2^,^3^,^4^,^5^,^6^,^7^,^8^. Several factors, such as different length of delays, payoff size, and payoff probability, may contribute to the inconsistent findings from previous studies. Hypothetical decisions have usually been associated with large payoffs while real decisions are typically made with small payoffs^8^. Moreover, for most previous DDT studies, real monetary rewards are not awarded in full; usually only one or a few selected responses are awarded as opposed to every trial. For example, participants were awarded about 1 reinforcer for every 90 choices in one study^2^, and were awarded 1 for every 412 and 1 for every 15 in two experiments in another study^4^. In the real reward condition of the present study, participants were awarded the exact amount of monetary rewards they won for the balloons, thus eliminating confounding factors associated with payoff size or probability.

In addition to behavioral differences, ERP data further revealed differential brain responses to negative feedback during the BART with real monetary rewards compared to hypothetical rewards. Specifically, a larger FRN was observed after balloon explosion during risk taking with real rewards. This finding may be interpreted by the prediction error in reinforcement learning theory. Numerous ERP studies on decision-making have consistently shown that the FRN is typically elicited by feedback stimuli associated with unfavorable or unexpected outcomes^23^,^24^,^25^,^26^,^29^,^30^,^31^,^32^,^33^,^34^. Larger FRN was also reported when strong expectancy was violated^35^. Compared to hypothetical monetary rewards which have no utility in the real world, real monetary rewards are directly related to individual wealth, status, economic interests, power and happiness. Consequently, real monetary rewards may enhance participants’ sensitivity to recent feedback, increase effort to formulate expectancy, and heighten the desire to win. Therefore, when subjects were confronted with a negative outcome (balloon explosion and money loss) during the real monetary reward condition, they might experience a greater sense of expectancy violation and prediction error during the real monetary condition compared to the hypothetical monetary condition, which led to a larger FRN.

Our findings are also consistent with the view that the FRN may reflect the evaluation of motivational and emotional consequences of decision outcomes^36^,^37^. FRN appears to be modulated by socio-emotional states and the degree of taking personal responsibility for performance^38^,^39^. Previous studies have shown that arousal level is increased after a loss compared to a gain of the same magnitude^40^, and that regret emotion is induced when individuals are aware that the outcome they obtained would have been better if they had chosen differently^41^. Greater regret has been reported when participants actually experienced a narrow loss compared to those who only imagined a narrow loss^42^. Thus, subjects in our study may have exhibited higher arousal and regret after loss trials during the real monetary condition compared to the hypothetical monetary condition, which elicited a larger FRN.

There are several limitations in our study. First, we did not examine the potential effects of different magnitudes of monetary rewards, which may influence differences in brain and behavior between real and hypothetical monetary rewards. Indeed, the previous BART study^20^ examined risk taking behavior during real and hypothetical monetary rewards in two conditions, a small reward (1 cent per pump) and a large reward (25 cents per pump). Participants exhibited significantly less risk taking behavior during the large real monetary reward condition than the small monetary reward condition. However, there were no differences in risk taking behavior when the rewards were hypothetical^20^. In the real monetary reward condition only, participants showed more risk averse behavior during the real monetary reward condition than the hypothetical monetary reward condition. This finding is consistent with behavioral results from the current study, in which we used a modified BART paradigm with a large reward (reward doubled after each pump, from 1 cent for the first pump to 2048 cent for the 12^th^ pump) and observed differences in risk taking behavior. Future studies are needed to further examine the effects of both small and large real and hypothetical monetary rewards on risk taking and decision making in the brain.

Second, we did not observe a relationship between FRN amplitudes and risk taking behavior on the BART. This null finding may be due to the limitations of our modified BART paradigm. Due to time constraints in our study, we decreased the maximal inflation capacity of each balloon trial from 128 pumps (original BART) to 12 pumps (our modified BART); thus we may have reduced the sensitivity of our task for measuring individual differences in risk taking performance. Our null correlation was consistent with another study which also shortened the number of inflation capacity to 20 pumps^26^,^27^. Future studies are needed to replicate the current study while maintaining the original BART parameters.

In summary, this study used ERP with the BART paradigm and demonstrated differential effects of real versus hypothetical monetary rewards on risk taking behavior and brain responses. These findings suggest a caution to decision researchers when drawing conclusions about real choices from hypothetical studies of intended behavior, especially when large monetary rewards are used. The results have important implications for future utility of real and hypothetical monetary rewards to modulate risk taking and decision making.

## Methods

### Subjects

Nighteen healthy undergraduate students (mean age = 19.6 ± 1.0 years, 10 males) participated in this study. All participants reported no history of neurological or psychiatric disorders or head trauma. We excluded participants if they reported that they had drunk alcohol or smoked tobacco during the two weeks prior to the test. The present study was approved by the Sun Yat-sen University ethics committee. The entire experimental protocol was carried out according to the approved guidelines, which were in accordance with the Declaration of Helsinki. All subjects provided written informed consent before enrollment and were compensated for participating in the study. They also received additional payment which was commensurate with earnings from the real monetary reward condition, but not the hypothetical monetary reward condition. Every participant ended up with earnings from 45 to 65 Chinese Yuan (about US $7 to $10).

### Tasks and Procedure

Participants sat comfortably in a chair and completed the BART with both real money and hypothetical rewards. Before the study, participants were instructed to maximize the amount of monetary reward on all tasks. They were told that only the earnings from the real monetary reward condition, but not the earnings from the hypothetical monetary reward condition, would be compensated to them after the study. With the exception of the facticity of monetary rewards, the two versions of the task were identical in all other respects.

**Figure 1:**
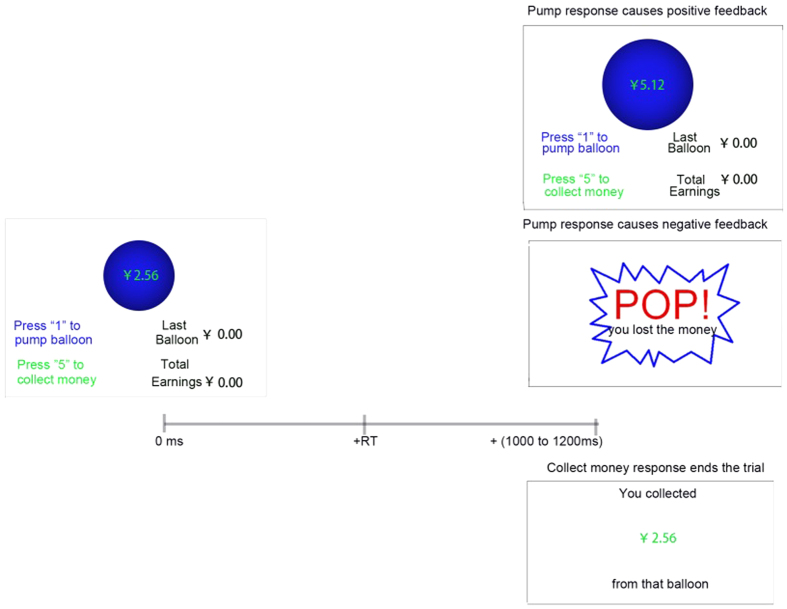
Schematic diagram for the BART. During the task, subjects are repeatedly given two options: press a button to continue inflating the balloon (the reward for each balloon increased with each inflation pump) or press another button to discontinue inflation. If subjects chose to stop inflation, they won the reward in the amount that the balloon was worth at the time they stopped inflation and the reward was added to their cumulative earnings. If subjects chose to continue inflation and the balloon exploded, they lost the reward for the current balloon, which was subtracted from the cumulative earnings as a penalty. Feedback stimuli were presented after a random delay (1–1.2 s) following subjects’ response.

**Figure 2:**
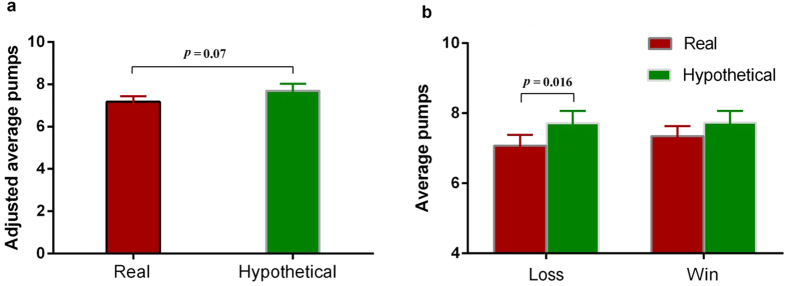
Behavior results from the BART with real and hypothetical monetary rewards. (**a**) When examining all trials, there was a trend that subjects made a smaller number of inflation pumps in the real reward condition than in the hypothetical reward condition. (**b**) During the balloon trials immediately following a loss, subjects pumped the balloon significantly less in the real reward condition than in the hypothetical reward condition after balloon loss trials. In contrast, during the balloon trials immediately following a win, there was no difference in number of inflation pumps between real and hypothetical reward conditions. Data presented as Mean ± SEM.

**Figure 3:**
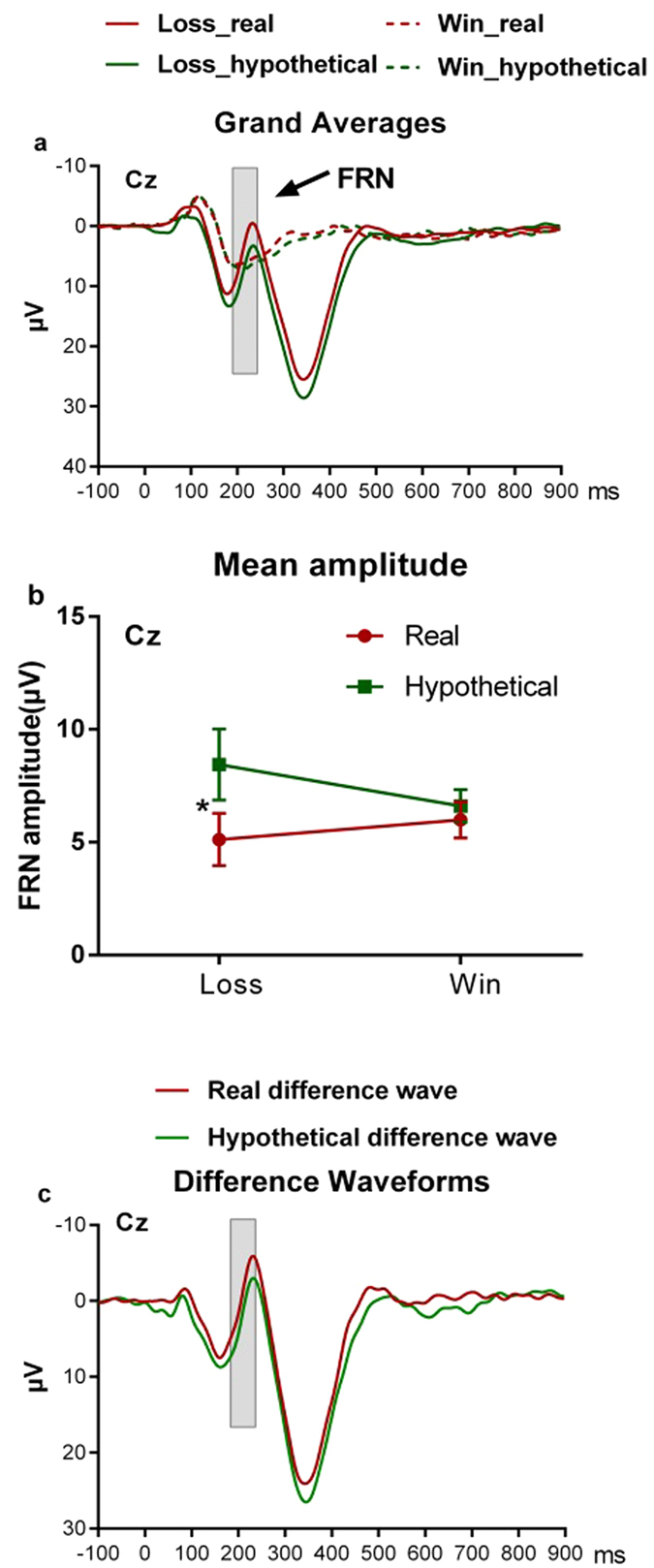
ERP waveforms, mean amplitudes, and difference waveforms. (**a**) Grand average ERP waveforms after the onset of loss and win feedback split by reward type. (**b**) Mean FRN amplitudes for loss and win feedback in real and hypothetical reward conditions. Data presented as Mean ± SEM; *p < 0.05. (**c**) Difference waveforms calculated by subtracting the win feedback-locked waveform from the loss feedback-locked waveform.

The BART paradigm (Fig. 1) was modified from previous studies^27^,^43^. During the task, subjects were asked to inflate a balloon that could either grow larger or explode. The balloon stimuli were blue spheres with radii that increased proportionally to the amount of money added. The visual angle of the first balloon is 4.5°, which increased by 0.3–0.54° after each inflation. Participants were repeatedly given two options: to press a button to continue inflating the balloon or to press another button to discontinue inflation and collect the reward for the current balloon. If participants chose to stop inflation, they won the reward for the current balloon, which was added to their cumulative earnings. If they chose to continue inflation and the balloon exploded, a silent balloon burst graphic with text indicating the loss of money was presented, and participants lost the reward for the current balloon, which was subtracted from the cumulative earnings as a penalty. The delay between the subject’s button press and feedback was randomized from 1 to 1.2 s.

In order to encourage participants to make multiple inflation attempts for a balloon, both real and hypothetical monetary rewards increased with the balloon size and the probability of explosion. The maximum number of inflation participants could make for each balloon was 12. The probability of the balloon bursting after first inflation was 1/11, after the second inflation was 1/10, and so on, until the 12^th^ pump. The monetary reward was 1 cent for the first balloon and doubled after each pump. The reward value corresponding to each balloon size and the cumulative earning for the task were explicitly displayed on the screen, whereas the exact probability of explosion associated with a given inflation were unknown to participants. Prior to the formal experiment, participants practiced several balloon trials for each condition and experienced both loss and win for the balloons. Each subject completed 100 balloon trials for the real monetary reward condition and 100 balloon trials for the hypothetical monetary reward condition. The order of the two conditions was counter-balanced between subjects. Similar to previous studies^21^,^43^,^44^, risk-taking behavior on the BART was measured by calculating the mean adjusted number of inflation pumps on the trials when the balloon did not explode.

### EEG Recording and Analysis

EEG was recorded from an array of 57 electrodes of the 10/10 system. Horizontal and vertical electroαoculogram (EOG) was also recorded. A common average reference was used for online EEG recording and EEG signal was off-line algebraically re-referenced to the average of the left and right mastoids. Electrode impedance was kept below 3 k Ω. EEG was amplified with a band pass of 0.1–100 Hz, digitized online at a sampling rate of 512 Hz, and then off-line filtered with a digital low-pass of 30 Hz. Each epoch of EEG was from 100 ms of pre-stimulus to 1000 ms of post-stimulus. Trials contaminated by eye blinks, eye movement or muscle potentials exceeding ±100 μv at any electrode were excluded before averaging. ERP were then averaged for all stimuli within each session. The baseline for ERP measurements was the mean voltage over the 100 ms pre-stimulus interval.

Based on the FRN literature^26^,^27^,^28^ and visual inspection of our ERP waveforms, we analyzed the FRN effects using the mean amplitudes of ERP responses to different trials in the 180–240 ms time window from the Cz location. A repeated measures ANOVA was used to examine the effects of reward type and feedback valence on FRN amplitudes. Greenhouse-Geisser correction for repeated measures was applied for statistical analysis.

## Additional Information

**How to cite this article**: Xu, S. *et al*. Real and hypothetical monetary rewards modulate risk taking in the brain. *Sci. Rep.* **6**, 29520; doi: 10.1038/srep29520 (2016).
